# Dulaglutide vs insulin in adolescents with thalassaemia-induced diabetes: effect on metabolism and atherogenesis (DIADEMA)

**DOI:** 10.1007/s00125-026-06835-x

**Published:** 2026-08-19

**Authors:** Amira Abdel Moneam Adly, Eman Abdel Rahman Ismail, Mohamed Salah Eldin Mohamed Abdel Kader, Manar Reda Sadeq, Nouran Yousef Salah

**Affiliations:** 1 Paediatrics Department, Faculty of Medicine, Ain Shams University, Cairo, Egypt; 2 Clinical Pathology Department, Faculty of Medicine, Ain Shams University, Cairo, Egypt; 3 Paediatrics Department, Faculty of Medicine, Misr University for Science and Technology, Cairo, Egypt; 4 Resident of Paediatrics, Ministry of Health, Cairo, Egypt; 5 The Academy of Scientific Research and Technology, Cairo, Egypt

**Keywords:** Adolescents, CGM-derived metrics, GLP-1 receptor agonists, Pancreatic reserve, Transfusion-dependent β-thalassaemia-induced diabetes

## Abstract

**Aims/hypothesis:**

Chronic iron overload represents a significant morbidity in transfusion-dependent β-thalassaemia (TDT), leading to various complications, including diabetes and CVDs. The glucagon-like peptide-1 receptor agonist dulaglutide has been approved for the treatment of type 2 diabetes in paediatrics. In addition, it was recently approved for the treatment of CVDs. Hence, this study aimed to assess the effect of dulaglutide compared with conventional insulin therapy on glycaemic metrics, pancreatic reserve, iron load and dyslipidaemia among adolescents with TDT-induced diabetes.

**Methods:**

The open-label, parallel-group, randomised-controlled DIADEMA trial took place at the Paediatric Haematology Unit and the Paediatric and Adolescent Diabetes Unit, Paediatrics Hospital, Ain Shams University, Cairo, Egypt. Eligibility criteria were adolescents with TDT-induced diabetes aged 10–18 years, uncontrolled on metformin. The 80 participants were randomly assigned based on a computer-generated randomisation sequence to receive either subcutaneous dulaglutide (0.75 mg weekly) or conventional basal-bolus insulin therapy. Participants were followed up for 24 weeks with assessment of fasting blood glucose, HbA_1c_, fructosamine, fasting C-peptide, continuous glucose monitoring (CGM)-derived metrics, haemolysis markers, serum ferritin and lipid profile. The primary efficacy endpoint was the change in the CV as an index for glycaemic variability from baseline to week 24. People assessing the outcomes were masked (blind) to group assignment.

**Results:**

Both groups were similar as regards baseline clinico-laboratory characteristics (*p*>0.05). After 24 weeks, dulaglutide resulted in a significant decrease in CV (from 39.45 ± 3.97% to 35.05 ± 4.30%; *p*<0.001), time above range, fasting blood glucose, HbA_1c_, fructosamine, total cholesterol and serum ferritin, with a significant increase in fasting C-peptide and time in range compared with baseline levels and with the insulin group (*p*<0.05). Although the glucose management indicator decreased in the insulin group after 24 weeks compared with baseline, the CV (from 39.85 ± 4.99% to 43.26 ± 5.75%; *p*<0.001) and time above range were significantly increased (*p*<0.001). The estimated standardised effect size for the between-group difference in the CV was 1.69 (95% CI 1.16, 2.22). No serious adverse event was reported.

**Conclusions/interpretation:**

The glucagon-like peptide-1 receptor agonist dulaglutide therapy was associated with greater improvements in glycaemic control and variability compared with insulin among adolescents with TDT-induced diabetes, without risk of hypoglycaemia or compromising safety. Moreover, dulaglutide might have a beneficial effect on iron overload, insulin secretion and lipid profile. These findings are exploratory and clinically provocative, warranting further investigation.

**Funding:**

This research received no specific grant from any funding agency in the public, commercial or not-for-profit sectors.

**Trial registration:**

ClinicalTrials.gov NCT07370922.

**Graphical Abstract:**

**Figure:**
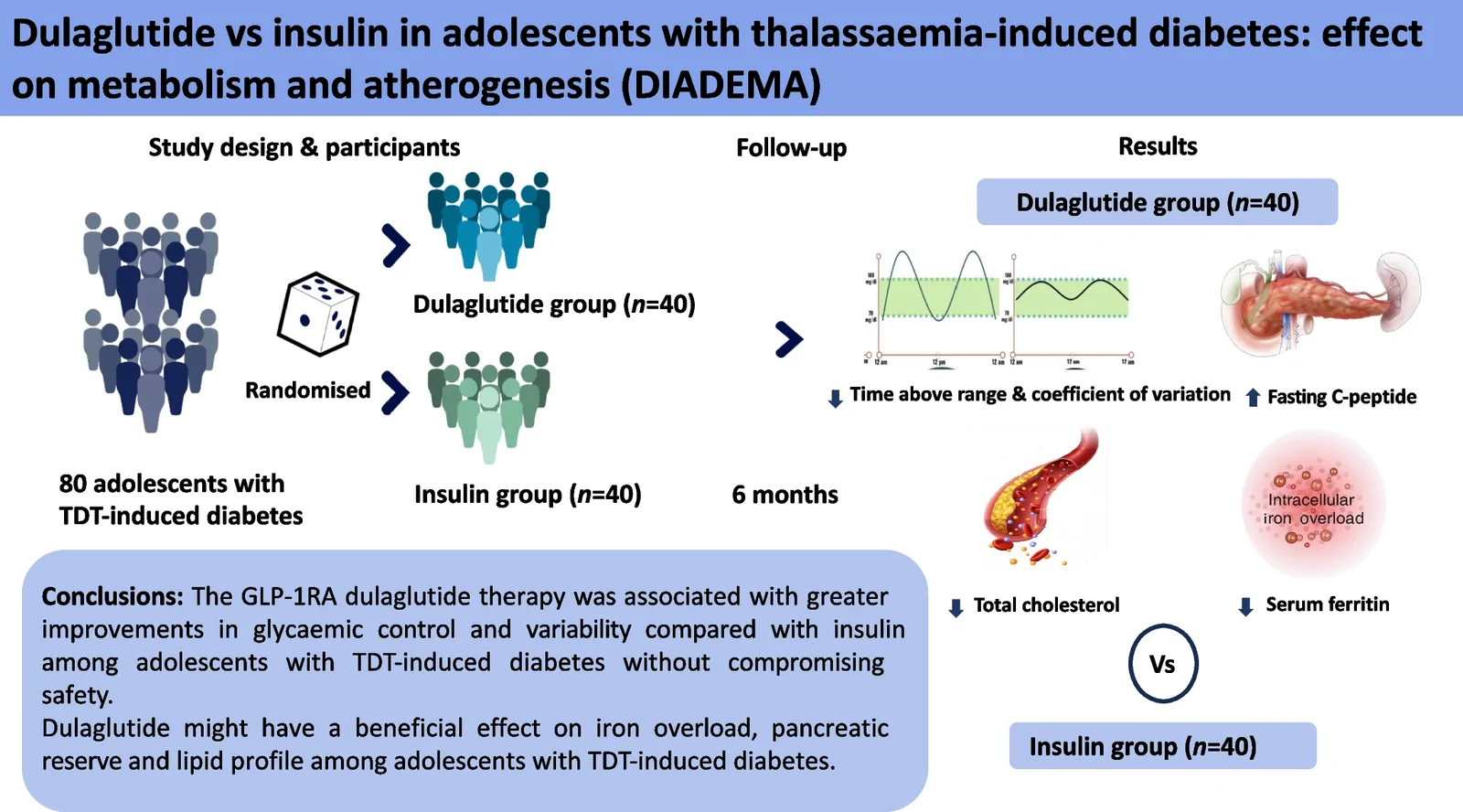

**Supplementary Information:**

The online version of this article (https://doi.org/10.1007/s00125-026-06835-x) contains peer-reviewed but unedited supplementary material.

**Figure:**
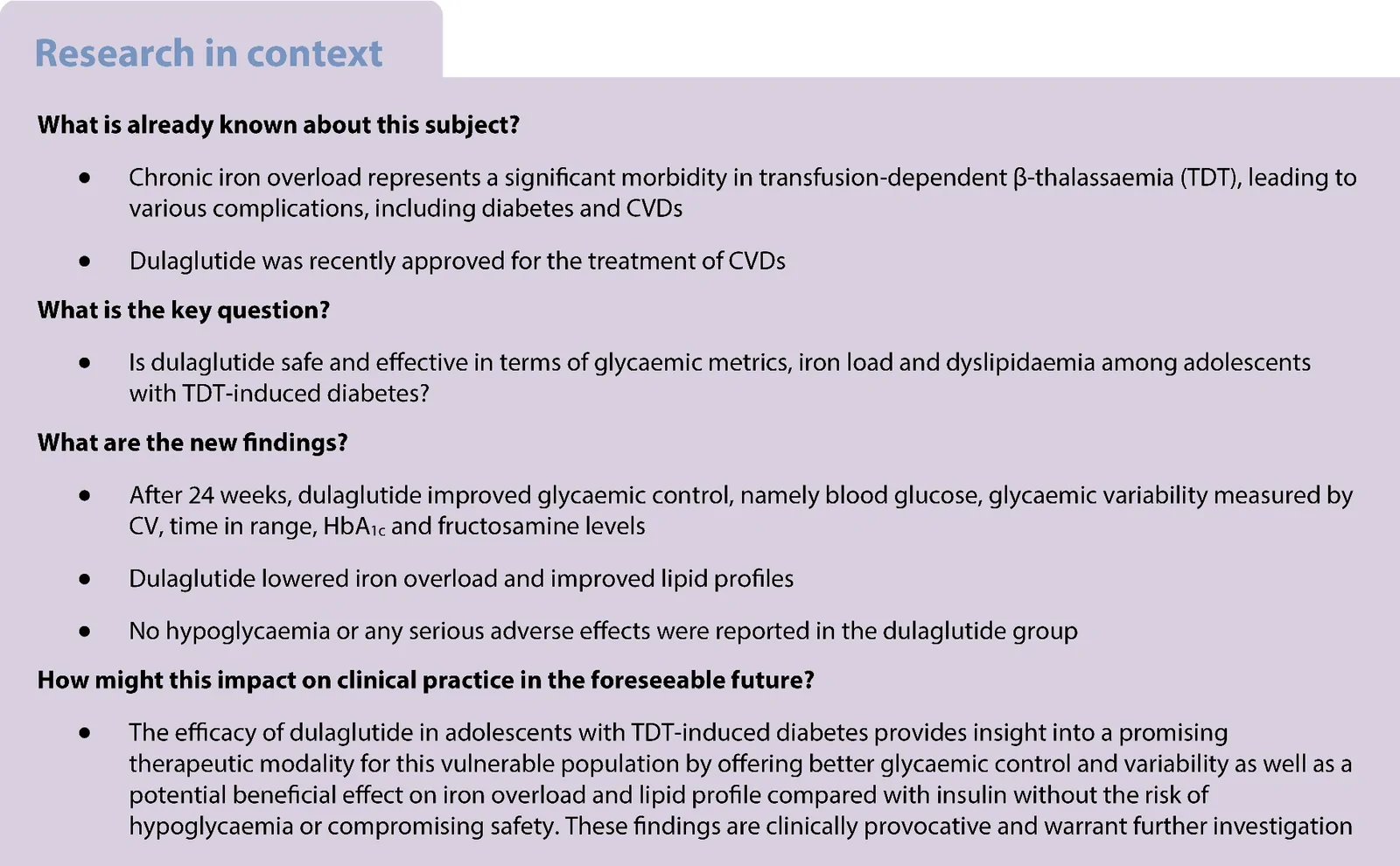

## Introduction

Transfusion-dependent β-thalassaemia-induced diabetes is a specific form of diabetes distinct from type 1 diabetes and type 2 diabetes but with similarities to both [1]. Iron overload disrupts the function of pancreatic beta cells, increases insulin resistance and precipitates diabetes [1, 2]. Advances in understanding the patho-mechanism and providing new therapeutic modalities relevant to this type of diabetes are crucial for better personalised therapeutic interventions and treatments [1].

Glucagon-like peptide-1 (GLP-1) is an incretin hormone that enhances glucose-dependent insulin secretion, slows gastric emptying and suppresses glucagon release. GLP-1 receptor agonists (GLP-1RAs), including the once-weekly dulaglutide, are transformative therapies initially developed for the management of obesity and type 2 diabetes. Recently, dulaglutide has been approved for the prevention of major adverse cardiovascular events and atherosclerotic CVD [3–5] as well as improving kidney outcomes [6] in people with type 2 diabetes. In addition, studies have shown a beneficial effect of GLP-1RAs in reducing serum and liver iron content in murine models of hereditary haemochromatosis [7, 8].

Given the beneficial effects of dulaglutide on glycaemic control in people with type 2 diabetes, this study aimed to assess the efficacy and safety of once-weekly dulaglutide compared with conventional insulin therapy among adolescents with transfusion-dependent β-thalassaemia (TDT)-induced diabetes and its impact on glycaemic metrics, pancreatic reserve, iron load and lipid profiles.

## Methods

### Study population

The open-label, parallel-group, prospective, randomised-controlled DIADEMA trial included 80 adolescents with TDT-induced diabetes aged 10–18 years, uncontrolled on metformin therapy, and recruited from the regular attendants at the Paediatric Haematology Unit and the Paediatric and Adolescent Diabetes Unit, Paediatrics Hospital, Ain Shams University. TDT was diagnosed according to the Thalassaemia International Federation guidelines based on clinical severity and transfusion requirements [1], while diabetes was diagnosed according to the International Society for Paediatric and Adolescent Diabetes (ISPAD) 2022 guidelines [9]. Sex and ethnicity were self-reported. All the enrolled participants were of the same race and ethnicity. Adolescents with other haemoglobinopathies, such as alpha thalassaemia or sickle thalassaemia; other disorders that may affect glucose homeostasis rather than TDT (e.g. autoimmune disease, collagen diseases, hypo- or hyperthyroidism, infections or tumours); a personal or family history of medullary thyroid carcinoma or multiple endocrine neoplasia type 2; and those who were taking any vitamins or food supplements 1 month before the study or participated in an investigational drug study within the 3 months preceding screening were excluded.

### Ethical considerations

The study was conducted in accordance with the Helsinki Declaration of 2008 after approval of the Institutional Review Board and the ethical committee of the Faculty of Medicine, Ain Shams University (approval number FMASU MS 794/2024). Written informed consent and assent were obtained from the participants and their legal guardians before enrolment in the study. Reporting of the study conforms to the Consolidated Standards of Reporting Trials 2010 statement. This trial was registered at ClinicalTrials.gov (NCT07370922).

### Sample size calculation

During protocol registration, before study start, the sample size was calculated. Using the STATA 10 program for sample size calculation and setting the alpha error at 5%, based on the results of Jódar et al [10], the analysis from AWARD-2 showed that the change from baseline to endpoint in glycaemic variability measured by the CV was −0.77 in the dulaglutide group compared with 0.01 in the insulin group, with a mean difference of −0.78 ± 1.50. According to this, a sample of 70 participants (35 in each group) was needed to achieve 80% power of the study. This was raised to 80 (40 per group) to account for a 10% dropout rate. No outcomes or analyses were modified after study initiation.

### Randomisation

**Fig. 1:**
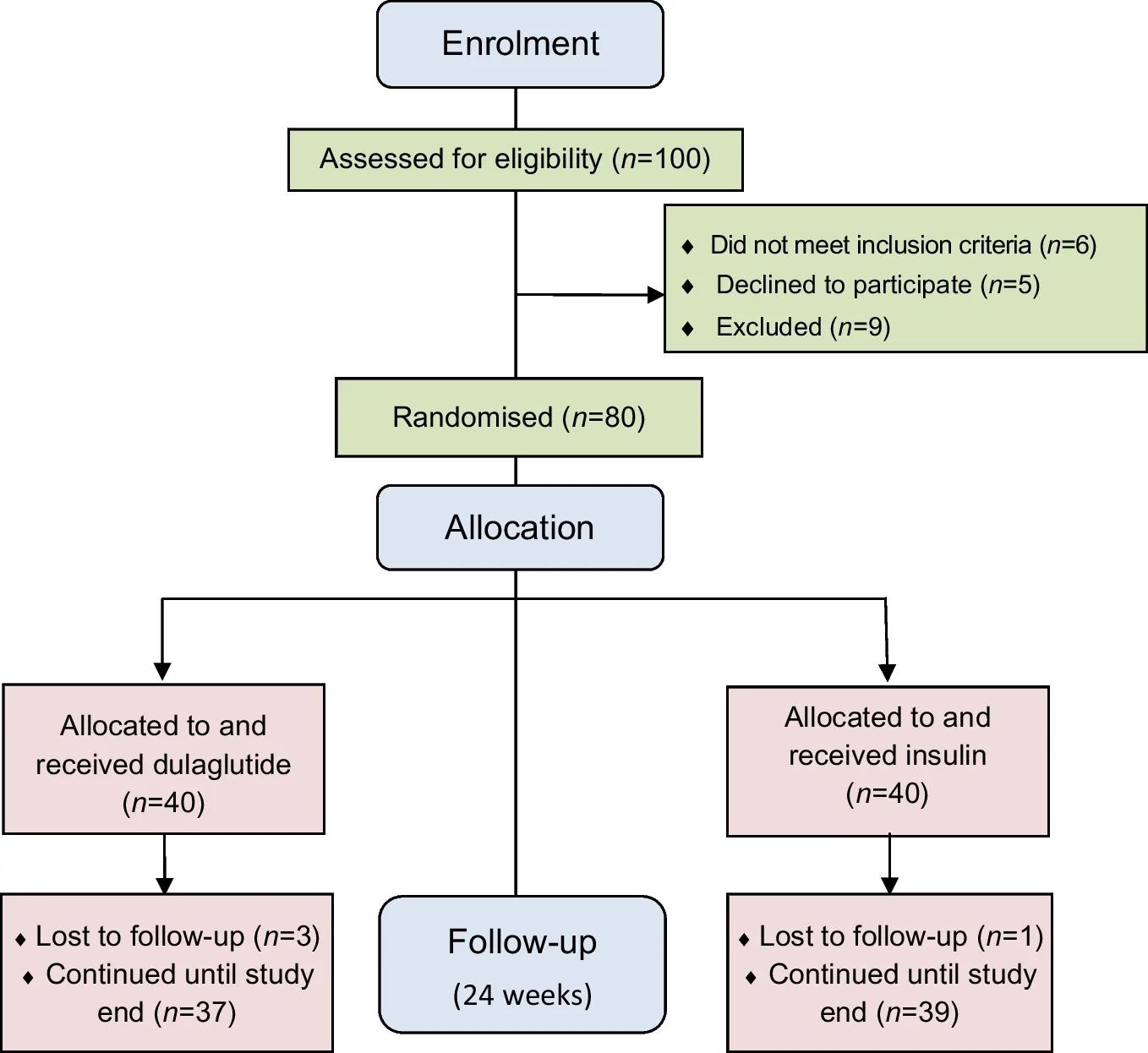
CONSORT flow diagram for the studied adolescents with TDT-induced diabetes

One hundred adolescents with TDT and diabetes were screened for eligibility; six patients did not meet the inclusion criteria, five declined to participate, nine were excluded, and 80 participants were enrolled (Fig. 1). Eligible participants were randomly assigned in a 1:1 manner based on a computer-generated randomisation sequence into two groups (40 participants per group). The intervention group received the GLP-1RA dulaglutide, Trulicity 0.75 mg/0.5 ml, in a dose of 0.75 mg s.c. once weekly for 24 weeks [11], while group II received conventional basal-bolus insulin therapy, including subcutaneous insulin degludec (Tresiba; Novo Nordisk, Copenhagen, Denmark) as the basal insulin and insulin aspart (NovoRapid, Novo Nordisk, Copenhagen, Denmark) as the mealtime insulin, titrated according to the ISPAD 2022 guidelines [12] (electronic supplementary material [ESM] Table 1). The insulin degludec dose was adjusted according to the mean previous weeks’ fasting blood glucose (FBG) readings. If one or more FBG values were missing, the dosage adjustment was performed based on the remaining values. Titration of insulin aspart was adjusted weekly by titrating the insulin-carbohydrate ratio according to the post-meal 2 h continuous glucose monitoring (CGM) reading. The mean total daily insulin dose of group II was 0.4 ± 0.31 U kg^−1^ day^−1^. All included participants were on mono-chelation therapy in the form of deferasirox (Jadenu; Novartis Pharma AG, Basel, Switzerland) at 17.5 mg kg^−1^ day^−1^.

### Study procedures

The enrolled adolescents with TDT-induced diabetes were subjected to a detailed medical history, laying stress on age, transfusion index (volume of transfused packed red cells in ml per kg body weight per year), type and dose of chelation therapy, diabetes duration, insulin dose and regimen, and history of splenectomy. Clinical examination included anthropometric measures: weight, height, waist circumference, and BMI with calculation of the SD score according to age and sex [13]. Waist circumference was measured midway between the top of the iliac crest and the lowest rib, with calculation of the SD score in comparison with normal references for age and sex [14]. Systolic and diastolic BP were measured using a mercury sphygmomanometer manually in the right arm three consecutive times while the patient was relaxed and seated, taking their mean values, and then the results were plotted on the age- and sex-appropriate percentiles [15].

CGM was recorded over 2 weeks at baseline and after 24 weeks using Freestyle Libre2 plus CGM (Abbott UK). Data was downloaded using the cloud-based Libre View system and interpreted according to the ISPAD 2022 Clinical Practice Consensus Guidelines [16]. Participants were instructed to avoid intake of paracetamol (Tylenol or Panadol), hydroxyurea and vitamin C (ascorbic acid) during the study to ensure the validity of CGM results.

Peripheral venous blood samples were collected in potassium ethylenediamine tetra-acetic acid (K₂-EDTA) (1.2 mg/ml) for a complete blood count and HbA_1c_ measurement. For chemical analysis, clotted samples were obtained, and serum was separated by centrifugation for 15 min at 1000 × *g*. Samples with gross haemolysis or lipaemia were discarded ‘to minimise the possibility of sample adventitious oxidation post-collection’. Urine samples were collected for assessment of urinary albumin excretion. At the time of blood sample collection, participants were free from any potentially confounding or interfering conditions, such as infections or fever.

Laboratory investigations included a complete blood count using Sysmex XN-1000 (Sysmex, Kobe, Japan); HbA_1c_ using D-10 (Bio-Rad, Marnes-la-Coquette, France); and serum ferritin on Cobas e411 (Roche Diagnostics, Mannheim, Germany). FBG, liver and kidney function tests, lactate dehydrogenase, and fasting lipid profile were measured using the Beckman Coulter AU480 chemistry analyser (Beckman Coulter Electronics, Hialeah, FL, USA). Fructosamine was assessed using a quantitative colorimetric assay on a Mindray BS-300 analyser (Diamond Diagnostics, Holliston, MA, USA), while fasting C-peptide was measured on Cobas e411 with an electrochemiluminescence technique (Roche Diagnostics, Mannheim, Germany). Urinary albumin excretion was collected using an early morning fasting urine sample as an albumin/creatinine ratio by the immuno-turbidimetric method (Cobas C111; Roche Diagnostics, Mannheim, Germany). The eGFR was calculated using the Chronic Kidney Disease Epidemiology Collaboration (CKD-EPI) equation [17]. The estimated glucose disposal rate was also calculated [18].

### Follow-up

All participants were closely and clinically followed up weekly through regular clinic visits for 24 weeks to receive dulaglutide (for the dulaglutide group), titrate insulin dose (for the insulin group), assess compliance and monitor any signs of potential adverse effects during the study period. Participants were instructed to refrain from substantial changes in their lifestyle habits throughout the study period.

### Study endpoints

The primary efficacy endpoint was the change in the CV% (as an index for glycaemic variability) from baseline to week 24. Key secondary efficacy endpoints were the change in HbA_1c_, time in range (TIR), FBG and fructosamine from baseline to 24 weeks among adolescents with TDT receiving the GLP-1RA dulaglutide in comparison with conventional insulin therapy. The safety endpoints were the occurrence of any adverse events throughout the study period. Any side effects related to GLP-1RA were reported including gastrointestinal symptoms (nausea, diarrhoea, vomiting, constipation, abdominal pain and dyspepsia), increased heart rate, allergy, injection site reactions and hypoglycaemia defined as time below range (TBR) <3.9 mmol/l (70 mg/dl) >4% or clinically important hypoglycaemia defined as TBR <3.0 mmol/l (54 mg/dl) <1% on CGM reading [19].

### Data management and analysis

Data were collected, revised, coded and entered into the Statistical Package for Social Science (IBM SPSS) for Windows, Version 27.0 (Armonk, NY: IBM Corp). The Kolmogorov-Smirnov test was used to examine the normal distribution of variables. A comparison between two groups with qualitative data was done using the χ^2^ test. A comparison between two independent groups with quantitative data and parametric distribution was done using an independent *t* test, while data with non-parametric distribution were analysed using the Mann-Whitney test. To identify within-group changes (before and after 24 weeks of intervention), paired-samples *t* tests were applied for quantitative parametric data and the Wilcoxon rank-sum test for quantitative data with non-parametric distribution. To determine the effects of dulaglutide vs insulin at study end on BMI, BP and haematological and biochemical variables including glycaemic outcomes at study end, analysis of ANCOVA was performed adjusted for age and disease duration as covariates. A *p* value <0.05 was considered significant in all analyses. The analyses were conducted based on the intention-to-treat principle. Missing values were imputed using the baseline observation carried forward method.

## Results

The DIADEMA trial was conducted on 80 adolescents with TDT-induced diabetes. They were 50 boys (62.5%) and 30 girls (37.5%) with a mean age of 12.25 ± 1.33 years (range, 10–15 years) and a mean diabetes duration of 7.59 ± 1.79 (range, 2–11 years). They were randomised into two equal groups to receive dulaglutide or conventional insulin therapy for 24 weeks. Both groups were similar as regards age, sex, pubertal status, diabetes duration, and baseline clinical and laboratory data (*p*>0.05). At the study’s end, four participants dropped out of the study because they did not attend follow-up visits (three in the dulaglutide group and one in the insulin group).

### Effect of dulaglutide on haematological parameters, liver enzymes and iron overload

Dulaglutide led to a significant decrease in serum ferritin compared with insulin therapy (*p*=0.044). Moreover, alanine aminotransferase was significantly lower after dulaglutide therapy compared with baseline levels and with insulin therapy (*p*<0.001). However, no significant difference was found as regards haemoglobin level or markers of haemolysis between both study groups (*p*<0.05; Table 1). Similar results were observed when the per cent change of haematological and biochemical parameters from baseline to study end was compared between the dulaglutide and insulin groups (Table 2).

**Table 1:** Comparison between the studied children and adolescents with TDT-induced diabetes on dulaglutide or insulin as regards clinico-laboratory data at baseline and at study end

Variable	Dulaglutide group			Insulin group			*p* value^b^
	Baseline	At 24 weeks	*p* value^a^	Baseline	At 24 weeks	*p* value^a^	
BMI SDS	−0.23 (−0.45–0.37)	−0.22 (−0.41–0.36)	0.678	0.26 (−0.15–0.59)	0.43 (0.19–0.81)	0.012	<0.001
Systolic BP, mmHg	103.32 ± 7.86	102.94 ± 5.35	0.728	102.11 ± 7.51	100.96 ± 4.74	0.401	0.084
Systolic BP, percentile	41.63 ± 8.09	42.93 ± 5.60	0.400	39.36 ± 7.22	41.27 ± 7.00	0.231	0.244
Diastolic BP, mmHg	64.55 ± 6.94	65.68 ± 5.22	0.098	62.29 ± 3.69	67.95 ± 4.44	<0.001	0.024
Diastolic BP, percentile	41.77 ± 8.17	42.48 ± 14.22	0.767	40.21 ± 5.94	46.54 ± 6.53	<0.001	0.105
Transfusion index, ml kg^−1^ year^−1^	327.04 ± 103.42	326.85 ± 103.10	0.552	320.15 ± 102.20	317.77 ± 103.09	0.081	0.695
Haemoglobin, g/dl	8.40 ± 1.29	8.70 ± 0.88	0.059	8.14 ± 1.13	8.38 ± 0.91	0.072	0.118
Serum ferritin, µg/l	3854 (2524–5027)	2736 (2209–3855)	<0.001	3413 (2579–5066)	3701 (2768–4286)	0.037	0.044
Indirect bilirubin, µmol/l	7.35 ± 1.14	8.17 ± 1.01	0.032	7.11 ± 1.11	8.12 ± 1.29	0.015	0.815
LDH, U/l	236.43 ± 77.21	230.34 ± 57.40	0.696	244.69 ± 61.39	251.30 ± 43.73	0.451	0.07
ALT, U/l	31.00 (20.41–38.11)	29.75 (19.75–35.75)	<0.001	30.00 (30.01–43.50)	43.01 (35.75–73.25)	0.002	<0.001
AST, U/l	17.25 (10.25–24.03)	18.75 (17.01–21.50)	0.342	21.02 (15.75–25.01)	28.02 (24.75–46.75)	<0.001	<0.001
Serum creatinine, µmol/l	62.78 ± 7.34	64.44 ± 7.38	0.948	62.81 ± 7.19	73.11 ± 7.84	<0.001	<0.001
eGFR, ml min^−1^ 1.73m^−2^	105.88 ± 9.11	109.95 ± 10.55	0.369	104.62 ± 8.09	95.81 ± 10.61	<0.001	0.001
UACR, mg/mmol	1.35 ± 0.18	1.34 ± 0.15	0.857	1.43 ± 0.19	2.01 ± 0.21	<0.001	<0.001
Triglycerides, mmol/l	1.26 ± 0.41	1.01 ± 0.18	0.025	1.12 ± 0.39	1.24 ± 0.44	0.715	0.068
Total cholesterol, mmol/l	3.88 ± 1.01	3.01 ± 0.91	0.005	3.51 ± 1.14	4.36 ± 1.03	<0.001	0.031
HDL-C, mmol/l	1.18 ± 0.08	1.78 ± 0.11	<0.001	1.24 ± 0.12	1.13 ± 0.14	0.012	<0.001
LDL-C, mmol/l	2.51 ± 0.51	2.01 ± 0.19	<0.001	2.34 ± 0.31	2.52 ± 0.11	0.021	<0.001

The data are presented as mean ± SD for parametric variables and as median (IQR) for non-parametric variables

ALT, alanine aminotransferase; AST, aspartate aminotransferase; HDL-C, HDL-cholesterol; LDH, lactate dehydrogenase; LDL-C, LDL-cholesterol; SDS, SD score; UACR, urinary albumin creatinine ratio

^a^*p* value was obtained from paired-samples *t* tests for parametric variables and Wilcoxon rank-sum test for non-parametric variables

^b^*p* value was obtained using ANCOVA

**Table 2:** Per cent change from baseline to study end of haematological and biochemical parameters in children and adolescents with TDT-induced diabetes on dulaglutide or insulin

Variable	Dulaglutide group	Insulin group	*p* value
Transfusion index, ml kg^−1^ year^−1^	−0.35 (−0.46 to −0.26)	−0.43 (−0.59 to −0.29)	0.140
Haemoglobin, g/dl	1.68 (−2.67 to 10.96)	4.04 (−1.41 to 10.21)	0.715
Serum ferritin, µg/l	−24.08 (−34.45 to −10.20)	−12.62 (−21.87 to 12.40)	0.004
Indirect bilirubin, µmol/l	19.21 (2.15–36.71)	17.86 (2.92–40.28)	0.165
LDH, U/l	13.84 (−23.61 to 33.41)	7.49 (−7.85 to 20.84)	0.744
ALT, U/l	−22.44 (−37.27 to 6.94)	23.83 (−4.07 to 125.28)	<0.001
AST, U/l	23.93 (−20.41 to 80.00)	63.04 (21.13–104.40)	0.003
Serum creatinine, µmol/l	2.91 (−5.97 to 18.23)	15.79 (8.12–29.79)	0.001
eGFR, ml min^−1^ 1.73 m^−2^	1.24 (−5.56 to 5.92)	−8.02 (−16.45 to 0.39)	0.006
UACR, mg/mmol	−0.89 (−24.18 to 11.21)	38.91 (1.24–53.67)	<0.001
Triglycerides, mmol/l	−17.21 (−29.46 to 1.74)	9.75 (−2.04 to 16.36)	<0.001
Total cholesterol, mmol/l	−20.16 (−30.19 to −6.58)	26.76 (6.76–31.14)	<0.001
HDL-C, mmol/l	39.4 (7.85–55.67)	−9.14 (−16.54 to 4.73)	<0.001
LDL-C, mmol/l	−18.19 (−27.69 to −9.14)	7.94 (1.87–11.19)	<0.001

The data are presented as median (IQR)

ALT, alanine aminotransferase; AST, aspartate aminotransferase; HDL-C, HDL-cholesterol; LDH, lactate dehydrogenase; LDL-C, LDL-cholesterol; UACR, urinary albumin creatinine ratio

### Metabolic effects of dulaglutide

Table 1 shows a significant decrease in total cholesterol and LDL-cholesterol with increased HDL-cholesterol in the dulaglutide group at the study’s end compared with the insulin group (*p*<0.001). Of note, dulaglutide administration did not lead to a significant increase in BMI after 24 weeks (*p*=0.678), unlike that observed in the insulin group (*p*<0.001).

### Effect of dulaglutide on glycaemic metrics and pancreatic reserve

**Fig. 2:**
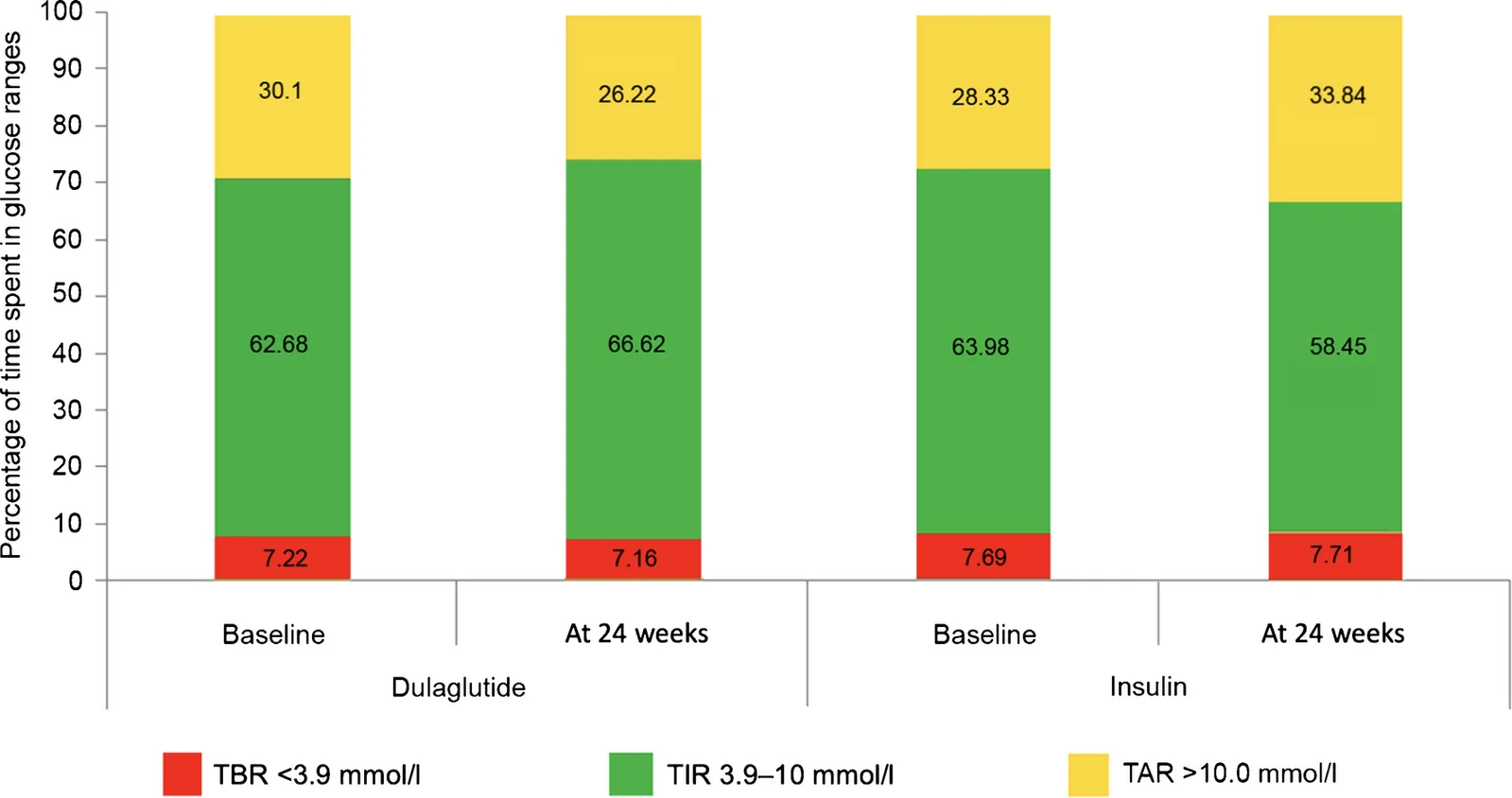
The percentage of time spent in glucose ranges among the studied adolescents with TDT-induced diabetes on dulaglutide or insulin at baseline and study end

As shown in Table 3, there was a significant decrease in FBG, HbA_1c_, fructosamine, glucose management indicator (GMI), CV and time above range (TAR) >10.0 mmol/l (>180 mg/dl) in the dulaglutide group after 24 weeks compared with the insulin group (*p*<0.001). This was associated with a significant increase in the TIR 3.9–10.0 mmol/l (70–180 mg/dl) (*p*<0.001; Fig. 2). Importantly, there was no significant change in the TBR <3.9 mmol/l (<70 mg/dl) in both groups at the study end (*p*>0.05). In addition, dulaglutide administration led to a significant increase in the fasting C-peptide (*p*=0.005) and estimated glucose disposal rate (*p*<0.001) at 24 weeks compared with insulin. Although GMI was decreased in the insulin group after 24 weeks compared with baseline levels, the CV and TAR >10.0 mmol/l were significantly increased (*p*<0.001). The estimated standardised effect size for the between-group difference in the CV was 1.69 (95% CI 1.16, 2.22).

**Table 3:** Glycaemic profile among the studied children and adolescents with TDT-induced diabetes on dulaglutide or insulin at baseline and at study end

Variable	Dulaglutide group				Insulin group				*p* value^b^
	Baseline	At 24 weeks	Per cent change	*p* value^a^	Baseline	At 24 weeks	Per cent change	*p* value^a^	
FBG, mmol/l	2.15 ± 0.31	1.84 ± 0.27	−15.39 (−19.13 to −13.47)	<0.001	2.13 ± 0.32	2.12 ± 0.25	−0.45 (−2.14 to 1.16)	0.816	<0.001
HbA_1c_, mmol/mol	70.12 ± 7.18	50.02 ± 6.57	−27.16 (−38.11 to −12.68)	<0.001	74.52 ± 7.94	65.11 ± 7.11	−11.78 (−16.75 to −4.72)	<0.001	<0.001
HbA_1c_, %	8.54 ± 1.06	6.77 ± 0.91	−12.97 (−31.86 to −11.94)	<0.001	8.97 ± 1.52	8.00 ± 1.58	−11.89 (−13.07 to −9.32)	<0.001	<0.001
Fructosamine, μmol/l	372.88 ± 37.68	341.06 ± 31.13	−7.11 (−18.25 to −3.47)	<0.001	376.97 ± 35.84	365.36 ± 38.76	−3.93 (−6.22 to −1.93)	0.321	<0.001
Fasting C-peptide, ng/ml	0.70 ± 0.19	0.95 ± 0.08	45.45 (9.32–66.67)	<0.001	0.81 ± 0.31	0.83 ± 0.21	14.91 (−16.05 to 56.78)	0.178	0.005
eGDR	5.12 ± 0.81	11.44 ± 2.11	98.7 (71.26–123.71)	<0.001	4.8 ± 0.91	7.6 ± 1.8	41.21 (34.11–58.42)	<0.001	<0.001
GMI, eA1C mmol/mol	73.04 ± 8.12	52.21 ± 7.34	−28.74 (−32.48 to −12.64)	<0.001	76.84 ± 8.57	65.32 ± 7.48	−13.94 (−19.11 to −5.68)	<0.001	<0.001
GMI, eA1C %	8.83 ± 0.93	6.85 ± 0.95	−19.21 (−31.72 to −12.90)	<0.001	9.22 ± 1.46	8.09 ± 1.52	−12.15 (−15.67 to −9.50)	<0.001	<0.001
CV, %	39.45 ± 3.97	35.05 ± 4.30	−10.08 (−17.42 to −2.98)	<0.001	39.85 ± 4.99	43.26 ± 5.75	8.70 (1.40–16.57)	0.001	<0.001
TIR 3.9–10.0, mmol/l	62.68 ± 5.11	66.62 ± 4.33	8.52 (−2.11 to 16.89)	<0.001	63.98 ± 5.79	58.45 ± 6.23	−5.95 (−12.52 to −0.35)	<0.001	<0.001
TBR <3.9, mmol/l	7.22 ± 1.21	7.16 ± 0.74	−4.65 (−15.13 to 10.47)	0.685	7.69 ± 1.99	7.91 ± 2.81	−18.06 (−25.29 to −5.67)	0.511	0.925
TAR >10.0, mmol/l	30.1 ± 5.27	26.22 ± 4.24	−17.09 (−31.18 to 5.78)	0.001	28.33 ± 5.39	33.84 ± 4.39	15.15 (7.19–31.78)	<0.001	<0.001

The data are presented as mean ± SD for parametric variables and as median (IQR) for non-parametric variables. The per cent change is presented as median (IQR)

^a^*p* value was obtained from paired-samples *t* tests

^b^*p* value was obtained using ANCOVA

eA1C, estimated A1C; eGDR, estimated glucose disposal rate; GMI, glucose management indicator

### Dulaglutide safety profile

As regards the adverse effects of therapy, 10 participants in the dulaglutide group reported gastrointestinal manifestations in the form of nausea, vomiting and abdominal distension. These manifestations were mild and waned by the third week of administration, and therefore, they did not necessitate the stoppage of treatment. As regards the insulin group, four participants had gastrointestinal manifestations, and 16 had hypoglycaemia, but none had clinically important or severe hypoglycaemia, and none had stopped treatment.

## Discussion

It has been reported that gut-derived peptide hormones (incretins), including dipeptidyl peptidase-4 inhibitors and GLP-1RAs, are beneficial in diabetes control in adults with TDT alone or in conjunction with metformin [2]. In addition, the International Consortium for Evidence-based Treatment of Type 2 Diabetes-Asia (ICET-A) Network recommended GLP-1RAs and dipeptidyl peptidase-4 inhibitors for treating basal and postprandial hyperglycaemia in thalassaemia-induced diabetes [20].

In the DIADEMA trial, dulaglutide treatment resulted in a significant improvement in glycaemic control and variability among adolescents with TDT-induced diabetes, as evidenced by reductions in FBG, HbA_1c_, fructosamine, GMI, CV and TAR with a concomitant increase in TIR 3.9–10.0 mmol/l. This was consistent with Houssarini et al [21], who reported that dulaglutide was associated with improved glycaemic control and a lower incidence of hypoglycaemia, supporting the metabolic advantage of dulaglutide in obese people with type 2 diabetes. Similarly, Ito and coworkers [22] compared intensive insulin therapy to a combination therapy of dulaglutide and long-acting insulin in 20 people with type 2 diabetes and found that both glycaemic control and variability were significantly improved, leading to a significant reduction in the number of self-injections needed.

The glycaemic improvement on dulaglutide could be attributed to its effect on insulin secretion promotion, enhancing insulin sensitivity, delaying gastric emptying and inhibiting glucagon production from pancreatic α-cells [23]. Interestingly, the increase in the TIR was not associated with an increase in the rate of hypoglycaemia as assessed by TBR. This is particularly important as it gives the GLP-1RA dulaglutide a merit over other glucose-lowering drugs that can increase the rate of hypoglycaemia. This could be because GLP-1 receptor activation in pancreatic beta cells promotes insulin release only when blood glucose levels are high, thereby avoiding hypoglycaemia. In addition, the inhibition of glucagon release from α-cells delays gastric emptying, leading to a more gradual absorption of nutrients, which further contributes to postprandial glycaemic stability [24].

In this regard, Huang et al [25] evaluated 236 people with type 2 diabetes and found that the dulaglutide group demonstrated a significant improvement in TIR and a reduction in TAR compared with the insulin group after 24 weeks of therapy, suggesting that adding dulaglutide enhances glycaemic stability and postprandial control without increasing hypoglycaemia risk.

Additionally, the dulaglutide group showed a significant increase in fasting C-peptide levels, suggesting enhanced endogenous insulin secretion. This is in line with Yoshiji and coworkers [26], who reported increased fasting and meal-induced C-peptide levels among people with type 2 diabetes on dulaglutide therapy. GLP-1RAs were found to increase the expression and activity of the transcription factor pancreatic duodenal homeobox-1 (PDX-1), which is specific for pancreatic beta cells; promote pancreatic beta cell proliferation and survival through the release of betacellulin; activate PI3K signalling and its downstream effectors and prevent beta cell apoptosis. Consequently, all these factors might lead to enhanced beta cell insulin secretion [27].

Notably, dulaglutide therapy did not result in weight gain among the studied participants with TDT-induced diabetes, unlike insulin, which resulted in a significant increase in BMI. Insulin is known to cause weight gain due to its anabolic effects, promoting fat storage and reducing glycosuria, suppressing lipolysis and fat oxidation, shifting metabolism toward energy storage, and stimulating appetite, as well as causing hypoglycaemic episodes that trigger compensatory eating [28].

The benefits of dulaglutide in the current study extend beyond glycaemic control, as it improved dyslipidaemia. A significant reduction in total and LDL-cholesterol was found at 24 weeks in the dulaglutide group. Tuttolomondo and coworkers [29] also reported a significant decrease in both total and LDL-cholesterol among people with type 2 diabetes on dulaglutide compared with controls after 9 months of follow-up. Similarly, a retrospective study on 55 adults with type 2 diabetes showed a significant decrease in LDL-cholesterol with marked improvement in glycaemic control, as evidenced by significant reductions in FBG and HbA_1c_ levels after 24 months of dulaglutide therapy [30]. This could be explained by previous studies that showed improved cholesterol metabolism upon the use of GLP-1RAs [31, 32].

It is worth mentioning that dulaglutide therapy led to a significant reduction in serum ferritin, reflecting a potential improvement in iron load. This is in line with Bain and colleagues [8] who evaluated 5522 people with type 2 diabetes and hereditary haemochromatosis of whom 1123 were on GLP-1RAs, including dulaglutide, and found that GLP-1RA use was significantly associated with lower serum ferritin concentrations compared with the control group. Moreover, preclinical murine models showed that GLP-1RAs markedly attenuated hepatic iron overload by downregulating the transferrin receptor and upregulating ferroportin [33]. Another study by Bozadjieva-Kramer et al [7] showed that GLP-1RA administration in a murine model of hereditary haemochromatosis decreased serum and hepatic iron levels, alleviating iron overload. In addition, a study by Melis and colleagues [34] showed that the use of GLP-1RAs reduced intestinal iron absorption among people with type 2 diabetes.

The beneficial effect of GLP-1RAs on iron homeostasis could be attributed to reduced iron absorption, altered hepatic iron metabolism and upregulating ferroportin, which helps reduce intracellular iron accumulation [34, 35]. Nevertheless, we only assessed serum ferritin, which is a variable surrogate marker that is influenced by transfusion burden and chelation therapy; therefore, the effect of dulaglutide on iron overload requires validation.

Notably, the dulaglutide group showed reductions in liver enzymes, reflecting improved hepatic function. In agreement with these results, Kuchay et al [36] studied 196 adults with type 2 diabetes treated with dulaglutide and demonstrated a significant reduction in alanine aminotransferase levels following dulaglutide therapy, suggesting improved hepatocellular integrity and reduced hepatic inflammation. These findings support dulaglutide’s potential role in ameliorating liver enzyme elevations commonly associated with metabolic dysfunction and non-alcoholic fatty liver disease. Furthermore, Shantaram et al [37] studied 84 adults with obesity and biopsy-confirmed hepatic steatosis who were treated with dulaglutide over 16 weeks. Participants showed a significant reduction in hepatic fat fraction, histological steatosis grade and hepatocellular ballooning with lowered liver enzymes, suggesting a weight-independent mechanism by which dulaglutide attenuates hepatic steatosis.

### Strengths and limitations

The current study has several strengths. It is the first to assess the efficacy of dulaglutide in adolescents with TDT-induced diabetes, providing insight into a promising therapeutic modality for this vulnerable population. Moreover, it assessed the effect of dulaglutide on pancreatic reserve, iron overload and lipid profile. Although we believe that our study participants were representative of the larger population of interest within the geographical location, the limitations of this study include the relatively small sample size and short CGM assessment period (2 weeks) as well as the lack of longitudinal endocrine profiling (e.g. IGF-1 and growth hormone). In addition, the open-label design, which was chosen because it was not feasible to mask the two treatments owing to the difference in the number of injection pens and the frequency of injections required for treatment, may have caused a risk of potential bias. This was minimised by blinding the endpoint assessors and using objective endpoints (CV, TIR, HbA_1c_ and fructosamine) as well as strict randomisation and allocation concealment to ensure group balance at baseline. Additionally, residual confounding due to chelation adherence, transfusion burden, treatment burden, adherence, behavioural responses and therapeutic complexity between once-weekly dulaglutide and basal-bolus insulin therapy may also have influenced comparative outcomes. Moreover, although a standardised insulin titration regimen was used, the complexity of optimising basal-bolus insulin therapy in adolescents with mixed insulin deficiency and insulin resistance requires cautious interpretation of comparative efficacy. Hence, future larger prospectively registered multicentre studies with extended follow-up are warranted to validate these findings and explore the mechanistic pathways.

### Conclusion

In the current study, treatment with the GLP-1RA dulaglutide was associated with greater improvements in glycaemic control and variability compared with insulin among adolescents with TDT-induced diabetes without risk of hypoglycaemia or compromising safety. This suggests that dulaglutide might have a beneficial role in lowering iron overload, enhancing insulin secretion and improving lipid profile. However, these findings are exploratory and clinically provocative, suggesting potential benefits that warrant confirmation in larger studies.

## Supplementary Information

Below is the link to the electronic supplementary material.

ESM Table 1 (PDF 74 KB)

## Data Availability

Data that support the study findings are available from the corresponding author, upon reasonable request, starting from the publication date and will remain accessible for a minimum of 5 years, in line with institutional policies via email. Access will be granted to qualified researchers to address research questions that are consistent with the scope of the original study and meet ethical and legal requirements.

## Abbreviations

CGM: Continuous glucose monitoring
FBG: Fasting blood glucose
GLP-1: Glucagon-like peptide-1
GLP-1RA: GLP-1 receptor agonist
GMI: Glucose management indicator
ISPAD: International Society for Paediatric and Adolescent Diabetes
TAR: Time above range
TBR: Time below range
TDT: Transfusion-dependent β-thalassaemia
TIR: Time in range
