# Supplementary material for: Dulaglutide vs insulin in adolescents with thalassaemia-induced diabetes: effect on metabolism and atherogenesis (DIADEMA)
Source: Diabetologia. 2026 Aug 19;69(11):3210–20. doi: 10.1007/s00125-026-06835-x (PMC13627196; doi:10.1007/s00125-026-06835-x)
Supplement: Supplementary file 1 [file 125_2026_6835_MOESM1_ESM.pdf]

**ESM Table 1:** Titration algorithm for weekly adjustment of basal and bolus insulin dosage

| **Previous weeks' mean FBG** | **Insulin degludec dosage adjustment (%)** | **Previous weeks' mean post-meal 2-hours CGM reading** | **ICR dose adjustment (%)** |
|---|---|---|---|
| >7.2 mmol/l (>130 mg/dl) | + 20% | >10.0 mmol/l (>180 mg/dl) | - 20% |
| 4.4-7.2 mmol/l (80 -130 mg/dl) | 0% | 5.0-10.0 mmol/l (90 - 180 mg/dl) | 0% |
| <4.4 mmol/l (<80 mg/dl) | - 20% | <5.0 mmol/l (<90 mg/dl) | + 20% |

FBG: fasting blood glucose; CGM: continuous glucose monitoring; ICR: insulin to carbohydrate ratio.
